# Hydrolysis of carotenoid esters from *Tagetes erecta* by the action of lipases from *Yarrowia lipolytica*

**DOI:** 10.1186/s40643-016-0131-7

**Published:** 2017-01-06

**Authors:** Abraham Figueiras Abdala, Alfonso Pérez Gallardo, Lorenzo Guevara Olvera, Eleazar Máximo Escamilla Silva

**Affiliations:** 1 Departamento de Ingeniería Química, Instituto Tecnológico de Celaya, Av. Tecnológico y A.G. Cubas s/n, 38010 Celaya, Gto Mexico; 2 Facultad de Química, Universidad Autónoma de Querétaro, Cerro de las Campanas s/n, 76010 Santiago de Querétaro, Querétaro de Arteaga Mexico; 3 Departamento de ingeniería Bioquímica, Instituto Tecnológico de Celaya, Av. Tecnológico y A.G. Cubas s/n, 38010 Celaya, Gto Mexico

**Keywords:** *Yarrowia lipolytica*, *Tagetes erecta*, Enzymatic hydrolysis, Carotenoid esters, Free lutein

## Abstract

The present study was conducted to evaluate the feasibility of enzymatic hydrolysis of carotenoid esters from *Tagetes erecta* using lipases from the yeast of *Yarrowia lipolytica*, with the aim of obtaining free lutein. The optimal concentrations of seven nutrients, considering the production of lipases relative to biomass (*Y*_p/x_) as the response variable, were determined in flask fermentations. In addition, we studied the effect on hydrolysis of growing *Y. lipolytica* in the presence of the oleoresin of the marigold flower in flask and stirred tank. Furthermore, hydrolysis of the oleoresin using the lipases from this microorganism was compared with the hydrolysis using lipases from *Rhizopus oryzae*. Cultured in the presence of marigold oleoresin, *Y. lipolytica* showed an increase in free carotenoids of 12.41% in flask and 8.8% in stirred tank, representing a fourfold and a threefold increase compared to the initial value in the fermentation, respectively. When lipases from the supernatant from both microorganisms were used for only 14 h hydrolysis experiments, a slight increase was achieved compared to a blank. We concluded that carotenoid esters of the oleoresin could not be completely hydrolyzed in 14 h by these lipases, but that growing *Y. lipolytica* in the presence of marigold oleoresin gives until fourfold production of free carotenoids in 72 h fermentations.

**Electronic supplementary material:**

The online version of this article (doi:10.1186/s40643-016-0131-7) contains supplementary material, which is available to authorized users.

## Background

Marigold flower (*Tagetes erecta*) is a plant capable of synthesizing various carotenoids of which, once extracted, lutein esters make up around 72% of them (Lim 2014). Studies aiming to increase the extraction yield of lutein esters have encouraged researches about the effect of enzymatic pretreatments to degrade cell walls and membranes in marigold flower (Barzana et al. 2002; Benitez-Garcia et al. 2014; Delgado-Vargas and Paredes-López 1997; Navarrete-Bolanos et al. 2004); however, this native plant of Mexico is mainly used as an ornament. The type and proportion of carotenoids of the plant depend on the variety being mostly lutein and zeaxanthin in yellow flowers, while for white flowers it is mostly lutein, zeaxanthin, β-cryptoxanthin and β-carotene (Benitez-Garcia et al. 2014). Studies showed that hydrolysis of zeaxanthin esters to achieve their free forms enhances the bioavailability of this carotenoid (Chitchumroonchokchai and Failla 2006). Unesterified lutein (free lutein) is the most interesting carotenoid since it is in great demand in the food, pharmaceutical, and cosmetic industries; its commercial market is expected to grow up to US $ 308 million in 2018 (Lim 2014; Lin et al. 2015a). Thus, carotenoid esters are usually subjected to saponification, which consists in making the oleoresin react with concentrated alcohol solutions of potassium or sodium hydroxide. The disadvantages of saponification are the degradation and isomerization of carotenoids, as well as the power costs and the need to implement safety measures for handling corrosive chemicals and waste products. In addition, the food industry is always looking for more natural alternatives for obtaining their products. Some studies have aimed to replace saponification with the aid of lipases from different microorganisms, but few studies have used marigold oleoresin (Zorn et al. 2003). After pretreatments with bile salts and protease from *Streptomyces griseus*, mature human milk was treated with lipases from *Candida rugosa* in order to hydrolyze retinyl esters to obtain free β-carotene and retinol from milk; however, this method still required further chemical hydrolysis (Liu et al. 1998). Hydrolysis of esters of astaxanthin was achieved using the non-specific cholesterol esterase which has been demonstrated to hydrolyze vitamins (Howles and Hui 2001; Jacobs et al. 1982). Carboxyl ester lipase (cholesterol esterase) achieved high activity for papaya and loquat extracts but low activity in incubations with paprika and marigold oleoresins. A porcine pancreatic lipase and a lipase from *Candida rugosa* was also tested and showed some activity on xanthophyll extracts (Breithaupt et al. 2002). Alkali labile carotenoids were hydrolyzed with a pig liver esterase converting astaxanthin dipalmitate to the monopalmitate and free astaxanthin (Aakermann et al. 1996). Astaxanthin, from *Haematococcus pluvialis* algal cell extracts, was effectively hydrolyzed by 5 fungal lipases in Tween 80-emulsified systems; under optimal conditions of pH, temperature, reaction time, and lipase dosage, free astaxanthin recoveries of 63.2% were achieved (Zhao et al. 2011). A process for enzymatic hydrolysis of carotenoid esters and other esters with aims of human and animal consumption has been presented as a patent; this process consists in the following: (1) incubate the esters with ester- cleaving lipases, and (2) appropriately isolate the resulting free forms (Flachmann et al. 2005). *Yarrowia lipolytica* is a yeast that has the potential industrial application of producing α-ketonic, acetic, citric, isocitric, pyruvic, and succinic acid; furthermore, it produces extracellular enzymes such as proteases and lipases of great industrial interest (Fletcher 2006; Gajdos et al. 2015); recently, metabolically engineered *Y. lipolytica* has been applied in fermentations where omega-3 eicosapentaenoic acid, a fatty acid with a wide range of health benefits, is produced by carefully balancing expression levels of pathway enzymes and modifying fatty acid and lipid metabolism (Xie et al. 2015). This microorganism has the ability to use fatty acids as a carbon source but the metabolism of these hydrophobic compounds is not yet fully understood; nevertheless, there are various proposed mechanisms in the literature for the use of fatty acids and alcohols by *Y. lipolytica* (Fickers et al. 2005; Fukuda and Ohta 2013; Hirakawa et al. 2009). It is considered a “safe to use” organism either as final product or as Yarrowia-derived product (Groenewald et al. 2014). Because of the safety of its use and its industrial interest, this work aims to explore the use of lipases produced by this microorganism to assess the lutein esters hydrolysis from an industrially obtained oleoresin into free lutein in flasks and 7 L stirred tank with 7 optimized nutrients. These results will also be compared with the use of lipases from *Rhizopus oryzae*.

## Methods

All of the reagents were reagent grade obtained from Sigma Chemical Co. (St. Louis, MO) and solvents were ACS grade unless otherwise specified.

### Biological material

The microorganism used for this study was the non-pathogenic yeast *Yarrowia lipolytica* strain CX39-74B (Strain number: ATCC 32339), which has a regulated dimorphism growth (Guevara-Olvera et al. 1993).

### Propagation of the strain of *Yarrowia lipolytica*

The strain was propagated by taking a sample by swab from a colony and streaking on YPD agar (yeast extract, peptone, dextrose; 1, 1, 2%). It was incubated for 24 h at 30 °C. Afterwards, 10 petri dishes with the yeast culture were stored as reserve at 4 °C. These reserve petri dishes were reseeded each month to prevent the aging of the culture. The preculture of *Y. lipolytica* in sterile saline solution was carried out in 500 mL Erlenmeyer flasks with 200 mL of YPD broth for 24 h at 30 °C and 250 rpm.

### Pigments

A sample of marigold oleoresin used in this work was donated by the company ALCOSA S.A. de C.V. (Celaya, Gto. Mexico); it had an average content of xanthophyll carotenoids (equivalent to all-trans-lutein) of 137.59 g/kg according to the official AOAC method 430.18, used by the company for quality control. It was stored under refrigeration at 4 °C in a sealed container until use.

### Analytical methods

#### Cell growth

The growth of cells in the culture broths was measured by the dry weight technique. A known volume of broth (5 mL) was filtered through a cellulose membrane (Merck ^®^) with a pore size of 0.22 μm previously dried to constant weight. Subsequently, the membrane was placed in an oven at 90 °C until a constant weight was obtained, and the weight difference was expressed as grams of dry cell weight per liter. In the case of the samples of culture media with oil, a known volume of broth was centrifuged in 50 mL tubes at 2600 rpm for 10 min; afterwards, the supernatant was discarded and the pellet was resuspended in distilled water. The process was repeated two more times. The final pellet was resuspended and filtered through the cellulose membrane. Cell growth was calculated by the weight difference.

#### Enzyme activity

The lipolytic activity was measured by the increase in absorbance at 401 nm caused by the release of ρ-nitrophenol as a result of the hydrolysis of ρ-nitrophenol palmitate by the action of the lipases present in the culture medium, using a Lambda 2 Perkin Elmer spectrophotometer. To perform the reaction, the sample of culture broth was first filtered through a cellulose membrane with a pore diameter of 0.22 μm to remove the suspended solids. This filtrate was the crude extract of the extracellular lipase enzyme. We added 2.5 mL of solution A (0.1 mM phosphate buffer solution, pH 7.8, and 100 mg of arabic gum per 90 mL) to a reaction tube, as well 0.2 mL of solution B (40.5 mg added of ρ-nitrophenol palmitate in 10 mL of isopropyl alcohol). This was placed in a water bath at 37 °C for 5 min. Subsequently, we added 0.3 mL of the crude extract and vortexed for 20 s. The mixture was placed again into the water bath for 10 min; after that time, the reaction was stopped with 2.5 mL of ethanol. The liquid was filtered through a nylon membrane with a pore size of 0.22 µm and the absorbance was read at 401 nm. Comparing the result with a calibration curve allowed us to determine the amount of released ρ-nitrophenol. Lipase units are defined as the µmols of ρ-nitrophenol released per minute per ml of sample.

#### Saponification

In order to carry out the saponification of the carotenoids contained in the oleoresin, we first extracted the pigments from 50 mg of the oleoresin using 30 mL of HEAT solution (hexane, ethanol, acetone, toluene; 10/7/6/7; v/v/v/v) in a 100 mL volumetric flask. Subsequently, we added 2 mL of methanolic potassium hydroxide, stirred for 1 min and heated to 56 °C for 20 min. The neck of the flask was connected to a condenser to prevent evaporation and loss of solvent. After the heating time had passed, the flask was left to cool and kept in the dark for 1 h. We then added 30 mL of hexane, stirred the mix, and filled the flask to the calibration mark with a solution of Na_2_SO_4_ (10%). Before performing any analysis, we allowed the mix to stand in the dark for 1 h. To assess the effect of saponification on pigments, we performed a thin layer chromatography. For this, we used silica gel plates (60 F254; 5 × 10 cm) for analytical chromatography. After drawing the reference line at 0.5 cm from the bottom, we placed 20 μL of each sample solution in the plates. The concentration of the unsaponified oleoresin solution was 0.5 mg/mL, while the concentration of the saponified solution was 1 mg/mL. The sample was eluted with increasing proportions of a mixture of hexane and acetone (80/20, v/v) within a closed chamber. Before the stains migrated to the upper end, the plate was removed and dried with nitrogen flow.

#### Extraction, characterization, and quantification of pigments

For the sample characterization according to its degree of esterification and amount of pigments, we used the AOAC official method 970.64 with the modifications introduced by Fletcher (2006). The difference with the original method is that the saponification step is omitted and larger volumes of elution solvents are used; also, the final dilution is greater than in the original method. With this method we were able to separate the fractions, those which had their xanthophylls quantified and those which had their degree of esterification measured. Thus, the pigments were classified as di-esters (E), monohydroxy pigments (MHP), free pigments or dihydroxy pigments (DHP), and residual pigments or polyols (RP). The content of xanthophylls was expressed as equivalents of trans-luteins. To extract the pigments, we dissolved 50 mg of oleoresin and 5 mL of the culture broth sample in 30 mL of HEAT using a 100 mL volumetric flask, adding 2 mL of methanol. After stirring, we left this mix in the dark for 1 h before conducting any analysis. Subsequently, the pigments were separated by open column chromatography. The column was 12.5 mm i.d. *X* 30 cm, with Teflon stopcock; the column was 2 mm i.d. and 10 cm long. The stationary phase comprised a mixture of silica gel–diatomaceous earth (1/1, w/w) activated by drying. The activation was carried out by heating in an oven for 24 h at 90 °C. The mixture was then cooled and stored in a desiccator before use. The column was packed with 7 cm of stationary phase and 2 cm of anhydrous sodium sulfate. After the column was packed and connected to a Buchner flask, an aspirator was connected to one of the ends of the flask to initiate the separation; the amount of sample of the extraction solution was 5 mL. We used four eluents: for carotenes and esters, hexane/acetone (96/4, v/v); for MHP, hexane/acetone (90/10, v/v); for DHP, hexane/acetone (80/20, v/v); for RP, hexane/methanol/acetone (80/10/10, v/v/v). The eluents were added in the same order until the corresponding fraction was recovered, which was then immediately transferred to the next solvent. Once the desired fraction was obtained, it was diluted with a known volume (25, 50 or 100 mL) of water, and its absorbance was read at 474 nm using the elution solvent as the blank. After determining the amount of pigments in each band or fraction, we calculated their percentage share of the total. In order to obtain ester-free lutein, we saponified a known amount of oleoresin, followed by open column chromatography in which the third band (corresponding to the DHP) was recovered. Lutein was identified by its spectral properties and its chromatographic behavior. No other tests were carried out because we were working with marigold oleoresin, in which lutein represents about 80% of the pigment content.

### Statistical method to investigate the factors effect involved in the production of lipases by *Y. lipolytica* on the composition of the culture medium

The effect of nutrients on the production of lipases was analyzed using a fractionated design 2^7−4^. The factors and levels of this design are summarized in Table 1. The design also involves a central experiment whose purpose is to determine the average of the levels studied as shown in Table 2. The variables that were measured are biomass (*X*), expressed as dry cell weight (g/L), and enzyme production, expressed as lipolytic activity in units per liter (U/L). We used these data to determine the fermentation kinetics of all of the experiments by sampling every 8 h for 72 h. The response variable of each treatment was the specific production of lipases or the biomass yield *Y*_p/x_ (U/g), which was obtained as the slope of the straight line fitted to the data of lipolytic activity (U/L) vs *X* (g/L), but only from the beginning to the end of the exponential growth phase.

**Table 1:** Factors and levels for the stirred flasks experiments

Factor	Compound	Levels	
		−	+
A	Olive oil	10 g/L	20 g/L
B	Yeast extract	3.0 g/L	5 g/L
C	KH_2_PO_4_	1.0 g/L	2 g/L
D	MgSO_4_·7H_2_O	1.4 g/L	2 g/L
E	NaCl	1.0 g/L	2 g/L
F	CaCl_2_	0.8 g/L	2 g/L
G	Trace elements solution^a^	2.0 mL	4 mL

^a^Each 100 mL contains: 50 mg of boric acid; 4 mg of CuSO_4_; 10 mg of KI; 20 mg of FeCl_3_; 20 mg of MnCl_2_; 20 mg of NaMoO4; and 40 mg and ZnSO_4_

**Table 2:** Treatments of the fractionated design with a central point

Factor		A	B	C	D	E	F	G
No.	Design coordinate	G/L						ml/L
1	−−++−−+	10	3	2	2	1	0.8	4
2	+++++++	20	5	2	2	2	2	4
3	−++−+−−	10	5	2	1.4	2	0.8	2
4	−+−+−+−	10	5	1	2	1	2	2
5	−−−−+++	10	3	1	1.4	2	2	4
6	+−−++−−	20	3	1	2	2	0.8	2
7	++−−−−+	20	5	1	1.4	1	0.8	4
8	0000000	15	4	1.5	1.7	1.5	1.4	3
9	+−+−−+−	20	3	2	1.4	1	2	2

+ and − represent high and low level, respectively, for factors ABCDEFG. 0 stands for a medium level which results from the average of the maximum and minimum value

The fermentations of the design were carried out in stirred flasks containing 200 mL of culture medium in duplicate. The temperature was 30 °C and the stirring speed was 200 rpm; Tween 20 (0.01%) was added to each flask to improve the emulsification of the oil. The pH was adjusted to 6.0 with sodium hydroxide at the start of the fermentation. The inoculum consisted of a preculture of *Y. lipolytica* in YEPD medium, of which we added a volume corresponding to 10% of the total fermentation volume. The cells of this culture were harvested in the period between the first third and the end of the exponential growth phase. We took samples every 8 h and we measured the enzymatic activity of lipases and the biomass. These measurements were performed in triplicate. The growth kinetics and the enzyme production of each data point were estimated from the average of the results of these three measurements for every treatment (data not shown). Data analysis was performed using JMP statistical software v.5.0.1.2 (SAS Institute 2015) to determine which factors influence the production of lipases and how they do it.

### Lipase production in stirred tank

In order to carry out the fermentations in a more controlled environment, we used a 7 L stirred tank bioreactor (Applikon, The Nederlands). The fermenter conditions were as follows: 4 L working volume, aeration of 0.8 vvm, stirring speed of 250 rpm, temperature of 30 °C, and pH 6. We inoculated with the strain propagated in YEPD medium using a volume equal to 10% of the working volume. The cells were harvested in the period between the first quarter and the end of the exponential growth phase. Sampling was initially done at 8, 12, and 18 h, finishing with 8-h sampling from 24 to 72 h, measuring lipase enzymatic activity and the biomass. Experiments were done in triplicate and expressed as the mean (SD) (standard deviation).

### Fitting the cell growth kinetics of *Y. lipolytica* to a mathematical model

The data obtained on the kinetics of cell growth were fitted to the logistic equation of population growth described by Verhulst (Peleg et al. 2007) which, applied to microbial growth, takes the form of Eq. 1.

1 $$X\left( t \right) = \frac{{X_{0} e^{\mu t} }}{{1 - \left( {\frac{{X_{0} }}{{X_{{{ \hbox{max} } }} }}\left( {1 - e^{\mu t} } \right)} \right)}}$$

This logistic model equation was entered in Microsoft Excel 2007 and, using the solver tool, we obtained the fitted parameters *μ*, *X*_0_ and *X*_max_ that minimized the squared differences between experimental values and calculated values.

### Effect of including the oleoresin in the culture medium of *Y. lipolytica* on the hydrolysis of carotenoid esters

The culture of *Y. lipolytica* was performed in the presence of 5 g of marigold oleoresin. Experiments were conducted in flask and stirred tank. The oleoresin was added to culture broth that had been previously emulsified. The emulsion composition was water/oleoresin/tween 20 (78/20/2, v/v/v). The yeast was cultured in 500 mL Erlenmeyer flasks using a working volume of 200 mL. In the preparation of the culture medium, we considered the volume and dilution that would increase the emulsion of marigold oleoresin. The medium composition was based on the results of the experimental design carried out in flasks. The culture conditions were temperature of 30 °C, stirring speed of 250 rpm, and pH 6 (adjusted with NaOH at the start of the fermentation). The inoculum volume was 10% of the working volume. Samples were taken every 12 h, assessing the amount of total carotenoid pigments and the fractions representing MHP, DHP, and RP. Three independent experiments were carried out; the assessments were made in triplicate and expressed as mean (SD). The culture conditions for experiments carried out in stirred tank were working volume of 4 L, aeration of 0.8 vvm, stirring speed of 250 rpm, temperature of 30 °C, and pH 6 adjusted at the beginning of fermentation. Samples were taken every 12 h, assessing the amount of total carotenoid pigments and the fractions representing E, MHP, DHP, and RP. Three independent experiments were carried out; the assessments were done in triplicate and expressed as mean (SD).

### Comparison between the activity of the lipases from *Yarrowia lipolytica* and from commercial *Rhizopus oryzae* in the hydrolysis of carotenoids esters from the marigold flowers

Tests were made in flasks to compare the activity of lipases produced by *Y. lipolytica* in the emulsified oleoresin, and the activity of commercial lipases produced by *R. oryzae* (Fluka, BioChemika). *Yarrowia lipolytica* was cultured in a medium with a composition based on the results of the experimental design carried out in flasks. The supernatant was harvested during the phase of greater lipolytic activity (48 h); 50 mL of this was taken and filtered through cellulose membrane with a pore size of 0.22 μm. The filtrate was placed in a 250 mL Erlenmeyer flask, adding CaCl_2_ to a concentration of 20 mM. Immediately, afterwards, we added approximately 50 mg of emulsified oleoresin in 6 g of triton X-100. The flask was placed under stirring at 150 rpm and 30 °C for 14 h. The hydrolysis test performed with lipases produced by *R. oryzae* was carried out as follows: 50 mL of phosphate buffer solution 0.1 M at pH 7.9 was added to a 250 mL Erlenmeyer flask, together with 5 mL of a solution containing the enzyme dissolved in distilled water at a concentration of 4 mg/mL. We then added about 50 mg of emulsified oleoresin in 6 g of triton X-100. The flask was stirred at 150 rpm and 30 °C for 14 h. The blank was prepared with 50 ml of distilled water and approximately 50 mg of emulsified oleoresin in 6 g of triton X-100 under the same experimental conditions. In both experiments, once the reaction time was complete, the pigments were extracted and quantified according to AOAC official method 970.64 with the modifications introduced by Fletcher (2006), which were described above. Three experiments were conducted. The assessments were done in triplicate and a variance analysis was done at the end to detect differences between treatments.

## Results and discussion

### Effect of the factors involved in the production of lipases by *Y. lipolytica*

In order to stablish the best culture medium, we performed a kinetic study aiming to determine the time to achieve the maximum value of the enzyme activity and the growth curve for this microorganism. After the model was fitted to the experimental data, we performed the regression formerly described to obtain the specific production of lipase (*Y*_p/x_). Table 3 shows the model parameters and the *Y*_p/x_ values for the 18 experiments, where we can see that the highest *Y*_p/x_ was achieved in treatment 3.

**Table 3:** Experimental and fitted data for the experimental design 2^7−4^

Treatment	Design coordinate^d^	Lipolytic activity^a^	Biomass^a^	*μ* ^c^	Specific yield of lipases^b^
		U/L	*X*_max_ (g/L)	h^−1^	*Y*_p/x_ (U/g)
3	−++−+−−	1153.6	3.556	0.080	510.56
1	−−++−−+	944.5	4.972	0.122	254.24
2	+++++++	1029.06	4.589	0.123	301.39
2	+++++++	1229.4	4.179	0.124	375.68
3	−++−+−−	1273.4	3.428	0.086	507.64
4	−+−+−+−	1111.7	4.391	0.100	391.71
1	−−++−−+	1023.3	4.909	0.124	220.09
5	−−−−+++	945.4	4.181	0.126	287.19
6	+−−++−−	1018.0	4.819	0.149	253.20
7	++−−−−+	981.9	4.598	0.118	310.47
4	−+−+−+−	1121.2	4.345	0.097	338.53
5	−−−−+++	936.0	4.026	0.129	276.97
8	0	947.9	5.106	0.109	233.71
6	+−−++−−	1030.0	4.256	0.129	324.98
7	++−−−−+	1050.0	4.509	0.128	262.01
8	0	1100.4	4.521	0.094	313.86
9	+−+−−+−	805.1	5.425	0.122	174.16
9	+−+−−+−	888.3	4.594	0.157	284.31

^a^Measures at 40 h of culturing

^b^Computed through the linear regression of lipolytic activity (U/L) vs *X* (g/L)

^c^Computed through the logistic model

^d^+ and − represent high and low level, respectively, for factors ABCDEFG. 0 stands for a medium level which results from the average of the maximum and minimum value

**Fig. 1:**
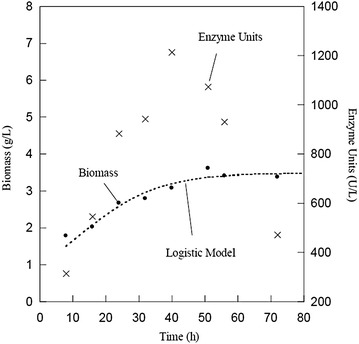
Kinetics of cell growth and lipases production of the best treatment of the experimental design 2^7−4^

Figure 1 shows the behavior of the culture of *Y. lipolytica* for treatment 3; it can be seen that the time needed for reaching maximum lipolytic activity (1213.5 U/L) is around 40 h. Similar lipolytic activity values were obtained by Pereira-Meirelles et al. (1997). It can also be seen that the maximum production of the enzyme coincides with the end of the exponential growth phase; for *Y. lipolytica,* this tendency may be explained by the extracellular lipase association with the cell membrane at the beginning of the growth phase and the release of lipases in the culture media at the end of the growth phase as confirmed in the literature by Western blot analysis (Fickers et al. 2004). However, the lipolytic activity decreased with the onset of the stationary growth phase which is probably due to the Zn^2+^ inhibitory effect since this ion is present in the trace elements solution (Fickers et al. 2013), caused by proteolysis as demonstrated in previous literature by adding a serine protease inhibitor (Pereira-Meirelles et al. 1997) or caused by a combination of both effects. The adjusted value of the *X*_max_ fitted for the model was 3.49 g/L. The obtained *μ* (0.083 h^−1^) indicated that growth was slow and that under these conditions the yeast would take longer to reach its maximum development. The specific production of lipase (*Y*_p/x_) used as a response variable was 508 U/g. Using the statistical program JMP, we estimated *Y*_p/x_ of 504 U/g resulting in a 0.8% of error between estimation and experimental value.

The analysis of variance for the 18 treatments of the design (Table 4) in combination with the effect of the factors shown in Table 5 allowed us to discern that olive oil, yeast extract, NaCl, and trace elements solution concentration produced significant differences (*p* < 0.05). The study of the effects of the concentrations of the selected nutrients allowed us to propose that the best composition of the culture medium of *Y. lipolytica* (the one with the greatest specific activity) was 10 g/L of olive oil, 5 g/L of yeast extract, 2 g/L of KH_2_PO_4_, 1.4 g/L of MgSO_4_, 2 g/L of NaCl, 0.8 g of CaCl_2_, and 2 mL of the solution of trace mineral nutrients.

**Table 4:** Analysis of variance of the design

Source	Degrees of freedom	Sum of squares	Mean square	*F*
Model	7	85,682.80	12,240.4	5.69*
Error	10	21,526.57	2152.7	
Total	17	107,209.37		

* Significant differences (*p* < 0.05)

**Table 5:** Effect of the factors selected for the experimental design 2^7−4^ on lipase production in terms of biomass (*Y*_p/x_)

Factor	Degrees of freedom	Sum of squares	*F*
Olive oil	1	15,645.63	7.39*
Yeast extract	1	53,274.41	25.16*
KH_2_PO_4_	1	2102.45	0.99
MgSO_4_	1	1464.78	0.69
NaCl	1	22,626.92	10.69*
CaCl_2_	1	2852.895	1.35
Solution of trace mineral nutrients	1	15,466.031	7.3*

* Significant differences (*p* ≤ 0.05)

A one-way direction ANOVA was studied and results are summarized in Table 6. From this, we can conclude that at least one of the means is significantly different from others. Moreover, a Tukey test was used in order to find means that are significantly different from each other (Table 7); we could determine that the mean of treatment 3 was significantly different (*p* < 0.05) from the means of the other treatments.

**Table 6:** One-way analysis of variance

Source	Degrees of freedom	Sum of squares	Mean square	*F*
Design	8	116,760.21	14595.0	7.36*
Error	9	17,846.22	1982.9	
Total	17	134,606.43		

* Significant differences (*p* ≤ 0.05)

**Table 7:** Tukey’s test for the means of the treatments

Treatment	Design coordinate	Groups^a^		Mean *Y*_p/x_ value
3	−++−+−−	A		509.10
4	−+−+−+−	A	B	365.12
2	+++++++	A	B	338.53
6	+−−++−−		B	289.09
7	++−−−−+		B	286.24
5	−−−−+++		B	281.88
8	0000000		B	273.78
1	−−++−−+		B	237.16
9	+−+−−+−		B	229.23

^a^Treatments with the same groups are not significantly different among themselves

### Confirmatory tests for lipase production in stirred flask

The fermentations in stirred flask under the optimal medium composition (see Additional file 1: Figure S1; Table S1) confirmed that the highest lipolytic activity occurs around 40 h. The *Y*_p/x_ was 481.2 U/g indicating that the model has a relative error of 4.8% for the confirmatory tests. The lipolytic activity obtained at 40 h was 1077 U/L, the maximum cell growth reached a value of 3.01 g/L, and the *μ* obtained was 0.127 h^−1^ was obtained.

### Results of the production of lipases in stirred tank

**Fig. 2:**
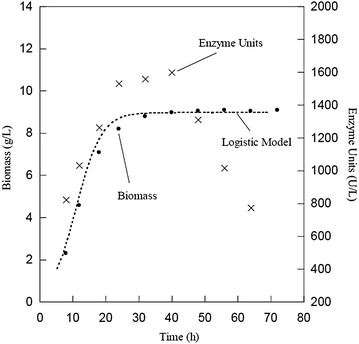
Kinetics of cell growth and lipase production in stirred tank

Table 8 shows the results of cell growth and enzyme production in the culture of *Y. lipolytica* in stirred tank. We obtained a maximum biomass of 8.9 g/L and *μ* of 0.236 h^−1^. The growth rate, higher than that obtained in the flask fermentations, indicated that the culture evolved rapidly. The maximum lipolytic activity of 1598 U/L was obtained at 40 h. Similar lipolytic activities were achieved by other authors (Pereira-Meirelles et al. 1997); however, an analysis of Fig. 2 indicated that there were no large increases in enzyme units between 24 and 40 h. Under these conditions, the microorganism achieved greater cell concentration and enzyme production than in stirred flask fermentations. Furthermore, the lipolytic activity decreased as the stationary phase continued beyond 40 h. Yield productivity in terms of biomass (*Y*_p/x_) was lower (113.8 U/g); thereby, the cell concentration increased. The increase in cell density and lipolytic activity compared to stirred flask experiments was attributed to the culture conditions in stirred tank, since we added stirring, aeration, and better temperature control. Alonso et al. (2005) pointed out the importance of oxygen in cell development; a higher content of oxygen accelerates the consumption of lipids, leading to increased biomass. In addition, stirring results in better dispersion of hydrophobic particles in the medium, enhancing their bioavailability. In contrast with our findings, Pereira-Meirelles et al. (2000) reported that the released lipase achieved its maximum concentration in the late stationary phase, and not in the beginning.

**Table 8:** Results of the kinetics of growth and lipase production in stirred tank

	*X*_0_^a^ g/L	*X*_max_^a^ g/L	Enzyme units (40 h) U/L	*µ* h^−1^	*Y*_p/x_ U/g
Average of three experiments	0.44	8.97	1598 (163.9)^b^	0.236	113.8^c^

^a^The data of *X*_0_ and *X*_max_ were calculated from the numerical solution of the logistic model applied to the average of three experimental runs

^b^The detected amount of enzyme units is shown as mean (SD) with *n* = 3

^c^The specific yield, or specific productivity, in terms of biomass (*Y*_p*/*x_) was calculated as described in the “Methods” section

### Characterization of marigold oleoresin

The results of thin layer chromatography showed that carotenoid esters were eluted faster than free carotenoids when silica gel was used as stationary phase. Table 9 summarizes the results of the fractionation by open column chromatography. This separation of carotenoid pigments, based on their degree of esterification, showed a predominance of di-esters, which represented 88.4% of the total. The monohydroxy and dihydroxy pigments represented 7.9 and 2.7%, respectively. The result of total xanthophylls (141.66 g/kg) was similar to the value of 137.59 g/kg reported by ALCOSA SA, differing by only 2.96%. It is noteworthy that, with their own extraction and identification protocols, Lin et al. (2015a) obtained samples containing free lutein equivalents of 0.44 g/kg of crude extract and Lin et al. (2015b) estimated 20 g/kg dry material.

**Table 9:** Results of the characterization of the marigold oleoresin by open column separation

Separated fraction	Xanthophylls^a,b^ g/kg	% of total carotenoids
Esters	125.23 (0.22)	88.4 (0.11)
MHP	12.53 (0.15)	7.92 (0.07)
DHP + RP^c^	3.9 (0.37)	2.68 (0.02)
Total xanthophylls	141.66	100.00

^a^Data are expressed as mean (SD) with *n* = 3

^b^Xanthophylls were quantified as equivalent to trans-lutein

^c^DHP + RP = free lutein + residual pigments

### Influence of the growth of *Y. lipolytica* cultured in oleoresin on the hydrolysis of carotenoid esters

**Fig. 3:**
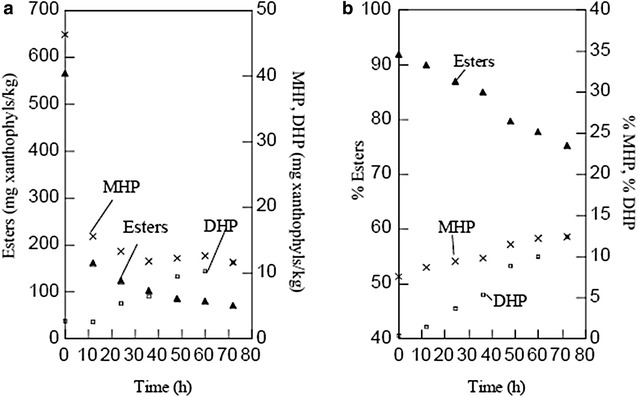
Changes in the esterification levels of carotenoids during fermentation in stirred flask. **a** Expressed as mg/kg of xanthophylls; **b** expressed in* percentages* of the total pigments

The hydrolysis of esters in stirred flask in the presence of oleoresin showed the behavior observed in Fig. 3a, b, i.e., a suddenly change in the content of esters and MHP during the first 12 h. This can be attributed to the aggregation of lipid particles—which at the beginning of the culture were emulsified and dispersed—since the enzyme cannot act properly if the substrate is not emulsified. Table 10 shows the average of three experiments on the behavior of the DHP during hydrolysis. It can be seen that, compared to the beginning of fermentation, there is an increase in the fractions of 4.62 and 12.41% after 48 and 72 h, respectively. This increase of 2.7–11.6 mg of DHP, representing a fourfold increase, is promoted by the aqueous solution, since DHP can disperse better in it than di-esters and MHP in the presence of stirring and emulsifying agents. The technical difficulties of using semi-solid fatty substances such as animal tallow for cultures of *Y. lipolytica* in stirred tank have been reported by Papanikolaou et al. (2002). These authors reported that the aggregation of lipid particles limits the proper performance of the cultures, and that higher stirring speeds are thus required to disperse these substrates in the medium.

**Table 10:** Increase of DHP due to the effect of culturing *Y. lipolytica* in stirred flask in a medium with marigold oleoresin, in different experiments

Experiment	% DHP^a^			% increase in free pigments^b^	
	Start (0 h)	Intermediate (48 h)	End (72 h)	Intermediate (48 h)	End (72 h)
1	0.34 (0.02)	4.3 (1.10)	16.04 (0.65)	3.96	15.7
2	0.48 (0.04)	3.6 (0.10)	10.75 (0.37)	3.12	10.27
3	0.5 (0.03)	7.3 (0.02)	11.76 (0.64)	6.8	11.26
Average	0.44	5.07	12.51	4.62	12.41

^a^Data are presented as mean (SD) with *n* = 3

^b^Calculated as the result of the difference between the mean percentages of DHP at the start and at the end of the fermentation

**Fig. 4:**
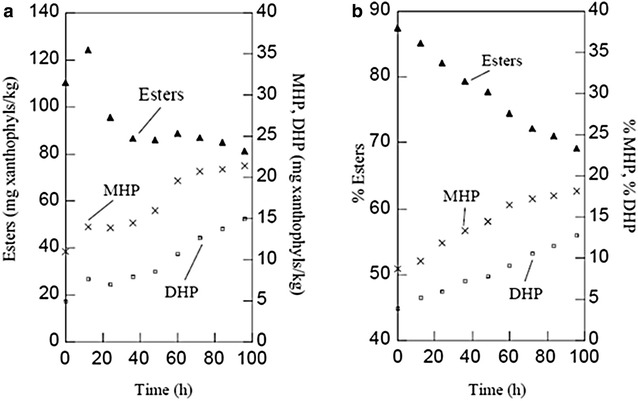
Changes in the esterification of carotenoids during fermentation in stirred tank. **a** Expressed as mg/kg of xanthophylls and; **b** expressed in their* percentage* of the total pigments

When *Y. lipolytica* was cultured in stirred tank in the presence of marigold oleoresin, we obtained an average of 8.8% of DHP after 96 h of culture. Table 11 shows the results of these three experiments. Figure 4 shows the average of 3 experiments of the changes in the degree of esterification of the carotenoids due to the effect of the growth of *Y. lipolytica* in stirred tank. It can be seen that the real change was from 4.9 to 15 mg of DHP, i.e., a threefold increase. There are two main differences with the experiments in flask. The first is that the contents of DHP and MHP are higher at the start of the culture probably due to the effect of mechanical stirring and to the coalescence of lipid particles, which may cause a decrease in pigment content at 48 h. The second difference is that after this period, mechanical stirring probably promoted the dispersion of the particles in the culture medium, and thus the concentration of DHP and MHO remained similar between 60 and 96 h. Finally, we should remark that the concentrations of MHP and DHP reached similar levels to those of flask experiments.

**Table 11:** Variation in the content of DHP due to the effect of growth of *Y. lipolytica* in a 7 L stirred tank in a culture medium with marigold oleoresin, in different experiments

Experiment	% DHP^a^				Increase in the % of free pigments^b^		
	Start (0 h)	Intermediate		End (96 h)	Intermediate		End (96 h)
		(48 h)	(72 h)				
					(48 h)	(72 h)	
1	3.8 (0.1)	7.5 (0.2)	10.7 (0.2)	12.6 (0.3)	3.7	6.9	8.8
2	3.7 (0.2)	8.4 (0.5)	10.2 (0.7)	11.7 (0.7)	4.7	6.5	8.0
3	4.1 (0.3)	7.2 (1.2)	10.5 (0.6)	13.7 (0.8)	3.1	6.2	9.6
Average	3.9	7.8	10.5	12.7	3.8	6.5	8.8

^a^Data are presented as mean (SD) with *n* = 3

^b^Calculated as the result of the difference between the mean percentages of DHP at the start and at the end of the fermentation

### Hydrolysis by lipases *Y. lipolytica* compared with commercial *Rhizopus oryzae*

Table 12 summarizes the results of the hydrolysis by *Y. lipolytica* and by *R. oryzae*. When the lipases secreted by *Y. lipolytica* were used for the hydrolysis of carotenoid esters, we obtained an average of 2.8% of free pigments out of the total amount of pigments; however, this represents a low increase in free carotenoids compared to a sample without lipase (blank). The grade of hydrolysis by *R. oryzae* was in accordance with previous literature (Zorn et al. 2003). The low yield was attributed to the fact the following facts: (1) the produced enzymes were not specific for the substrate used here, (2) the reaction was permitted to be proceeded for only 14 h, which was apparently not enough, and (3) reaction conditions of temperature, pH, aeration rate, and stirring speed have not been yet optimized. On this respect, it has been shown that other lipases show little or no activity in marigold extracts (Zorn et al. 2003). Even though it was minimal, the conversion rate was significantly different (*p* < 0.05) from the blank (1.8% of DHP) and the *R. oryzae* (2.6% of DHP) according to the variance analysis presented in Table 13.

**Table 12:** Increase in free pigments (DHP) in the hydrolysis of carotenoid esters from the marigold flower

Source of lipases^b^	Test	Mg/kg^a^			% of total pigments^a^		
		Esters	MHP	DHP	Esters	MHP	DHP
A	1	75.87 (0.02)	7.84 (0.06)	2.36 (0.02)	88.2 (0.04)	9.1 (0.06)	2.7 (0.03)
	2	83.65 (1.44)	8.03 (0.45)	2.53 (0.13)	88.8 (0.56)	8.5 (0.56)	2.7 (0.11)
	3	75.38 (0.06)	6.96 (0.07)	2.80 (0.07)	80.0 (0.98)	7.4 (0.10)	3.0 (0.04)
	Avg	78.30	7.61	2.56	85.7	8.3	*2.8*
B	1	76.47 (1.93)	5.93 (0.50)	2.23 (0.10)	90.3 (0.80)	7.0 (0.65)	2.6 (0.15)
	2	70.57 (0.20)	8.26 (0.10)	1.95 (0.06)	87.4 (0.15)	10.2 (0.09)	2.4 (0.07)
	3	70.61 (0.14)	7.94 (0.29)	2.23 (0.03)	87.4 (0.51)	9.8 (0.38)	2.8 (0.02)
	Avg	72.55	7.38	2.14	88.4	9.0	*2.6*
C	1	72.43 (0.13)	5.99 (0.21)	1.44 (0.08)	90.7 (0.29)	7.5 (0.24)	1.8 (0.09)
	2	72.39 (0.69)	6.05 (0.08)	1.51 (0.09)	90.7 (1.25)	7.6 (0.11)	1.9 (0.10)
	3	71.48 (0.06)	6.62 (0.08)	1.42 (0.11)	89.5 (0.50)	8.2 (0.18)	1.8 (0.13)
	Avg	72.10	6.22	1.46	90.3	7.8	*1.8*

^a^Data are presented as mean (SD) with *n* = 3

^b^A—*Yarrowia lipolytica*; B—*Rhizopus oryzae*; C—Blank

**Table 13:** Analysis of variance of the use of the hydrolysis of carotenoid esters from the marigold flower

Source	Degrees of freedom	Sum of squares	Mean square	*F*
Source of lipases	2	4.787	2.39	115.34*
Error	24	0.498	0.021	
Total	26	5.285		

* Significant differences (*p* ≤ 0.05)

## Conclusions

Given the great interest in natural free lutein for industrial purposes, it is highly important to find alternative methods for producing this xanthophyll such as the use of enzymes. The present work studied the enzymatic reaction of an industrially obtained oleoresin from marigold flower into free lutein to replace the chemical saponification using enzymes from *Y. lipolytica*. The yield of lipases by *Y. lipolytica* was, in descending order, mostly affected by the concentration of olive oil, yeast extract, sodium chloride, and trace mineral nutrients in the culture medium. Confirmatory experiments supported the statistical optimal values of the experimental design. With the addition of the oleoresin into the fermentation medium, the hydrolysis of carotenoid esters was achieved showing a strong increase in the free pigments fraction of the oleoresin (12.41%) in flask, and in stirred tank (8.8%) at 72 h, i.e., a fourfold and a threefold increase in mg of free carotenoids, respectively. Conducting 14 h hydrolysis tests with the enzymes from *Y. lipolytica* may not be enough time to transform most of the lutein esters; nevertheless, we obtained a poor conversion (2.8%) of the total oleoresin pigments to DHP against an experiment without lipases. Results were similar with lipases from *R. oryzae*. Therefore, carotenoid esters cannot be completely hydrolyzed with the lipases produced by *Y. lipolytica* in only 14 h, but culturing this microorganism in the presence of marigold oleoresin generates a high increase in the production of free carotenoids in 3-day fermentations. Further studies have to be carried out to optimize hydrolysis conditions and to better understand the system biology of this hydrolysis in order to evaluate the feasibility to achieve a better conversion.

## Abbreviations

DHP: free pigments or dihydroxy pigments; E: di-esters; HEAT: solvent solution (hexane, ethanol, acetone, toluene; 10/7/6/7; v/v/v/v); MHP: monohydroxy pigments; RP: residual pigments or polyols; SD: standard deviation; YPD: agar (yeast extract, peptone, dextrose; 1, 1, 2%)

### Nomenclature

*t*: fermentation time in h; *μ*: specific rate of growth in h^−1^; *X*_0_: initial biomass concentration in g/L; *X*_max_: final biomass concentration in g/L; *Y*_p/x_: production of lipases relative to biomass in lipase units U/L

## Additional file

**Additional file 1: Table S1.** Results of the confirmatory experiment for lipase production in stirred flask. **Figure S1.** Kinetics of cell growth and lipase production in the confirmatory tests carried out in stirred flasks.
