# Supplementary material for: Hydrolysis of carotenoid esters from Tagetes erecta by the action of lipases from Yarrowia lipolytica
Source: Bioresour Bioprocess. 2017 Jan 6;4(1):5. doi: 10.1186/s40643-016-0131-7 (PMC5236077; doi:10.1186/s40643-016-0131-7)

| **Table S1.** Results of the confirmatory experiment for lipase production in stirred flasks. | | | | | |  |
| --- | --- | --- | --- | --- | --- | --- |
| **Case** | ***X*_0_ ^a^**  **g/L** | ***X*_max_ ^a^**  **g/L** | ***µ***  **h-1** | **Enzyme units (40 hrs)**  **U/L** | ***Y*_p/x_ ^c^**  **U/g** |  |
| Average of the three confirmatory experiments. | 0.71 | 3.01 | 0.127 | 1070 (22.87) ^b^ | 481.2 |  |
| Estimation of the statistical program JMP. | --- | --- | --- | ---- | 504.29 |  |
| ^a^ The data of *X*_0_ and *X*_max_ were calculated from the numerical solution of the logistic model applied to the average of three experimental runs.  ^b^ The detected amount of enzyme units is shown as mean (SD) with n=3.  ^c^ The specific yield, or specific productivity, in terms of biomass (*Y*_p/x_) was calculated as described in the materials and methods section. | | | | | | |

**Figure captions of supplementary material**

**Figure S1.** Kinetics of cell growth and lipase production in the confirmatory tests carried out in stirred flasks


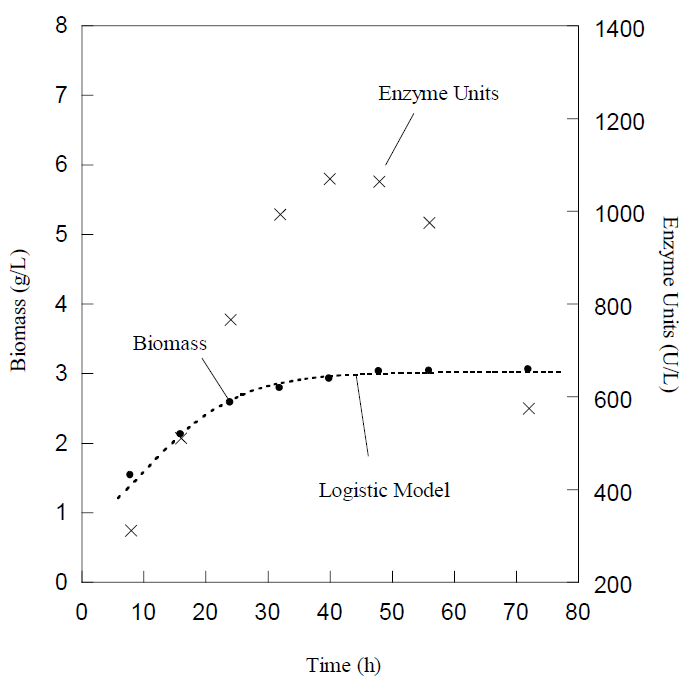

Supplement: Supplementary file 1 — Additional file 1: Table S1. Results of the confirmatory experiment for lipase production in stirred flask. Figure S1. Kinetics of cell growth and lipase production in the confirmatory tests carried out in stirred flasks. [file 40643_2016_131_MOESM1_ESM.docx]
